# Dietary Habits, Obesity, and Bariatric Surgery: A Review of Impact and Interventions

**DOI:** 10.3390/nu17030474

**Published:** 2025-01-28

**Authors:** Mădălina Maxim, Radu Petru Soroceanu, Vlad Ionuț Vlăsceanu, Răzvan Liviu Platon, Mihaela Toader, Ancuța Andreea Miler, Alina Onofriescu, Irina Mihaela Abdulan, Bogdan-Mihnea Ciuntu, Gheorghe Balan, Felicia Trofin, Daniel Vasile Timofte

**Affiliations:** 1 “Grigore T. Popa” University of Medicine and Pharmacy, Faculty of Medicine, Str. Universitatii, No 16, 700115 Iasi, Romania; madalynamaxim@yahoo.com (M.M.); petru.soroceanu@umfiasi.ro (R.P.S.); vlasceanu.vlad@yahoo.com (V.I.V.); platonliviurazvan@gmail.com (R.L.P.); Romania; mihaela.toaderr@gmail.com (M.T.); miler.ancuta@umfiasi.ro (A.A.M.); alina.onofriescu@umfiasi.ro (A.O.); bogdanmciuntu@yahoo.com (B.-M.C.); gheorghe-g-balan@umfiasi.ro (G.B.); dantimofte@yahoo.com (D.V.T.); 2 Department of General Surgery, County Clinical Emergency Hospital St. Spiridon, 700111 Iasi, Romania; 3 Department of Diabetes and Metabolic Diseases, Clinical Emergency Hospital St. Spiridon, 700111 Iasi, Romania; 4 Department of Medical Specialties I, “Grigore, T. Popa” University of Medicine and Pharmacy, 700115 Iasi, Romania; irina.abdulan@yahoo.com; 5 Department of Gastroenterology, Clinical Emergency Hospital St. Spiridon, 700111 Iasi, Romania; 6 Department of Preventive Medicine and Interdisciplinarity—Microboology, “Grigore T. Popa” University of Medicine and Pharmacy, Str. Universitatii no 16, 700115 Iasi, Romania

**Keywords:** obesity, eating behavior, bariatric surgery, weight loss, eating issues

## Abstract

Eating behavior encompasses the psychological, physiological, and environmental factors influencing food intake. Dysregulation in eating behavior, such as emotional eating, binge eating, or loss of satiety signals, contributes to excessive caloric intake and weight gain. These behaviors are often linked to hormonal imbalances, stress, or genetic predisposition. Obesity is a chronic, multifactorial disease characterized by excessive body fat accumulation, with a body mass index (BMI) ≥ 30 kg/m^2^ often used for diagnosis. It is associated with significant morbidity, including type 2 diabetes, cardiovascular disease, and obstructive sleep apnea. Pathophysiological mechanisms underlying obesity include insulin resistance, leptin dysregulation, and altered gut microbiota, which perpetuate metabolic derangements. Lifestyle interventions remain first-line treatment, but sustained weight loss is challenging for many patients. Bariatric surgery is a therapeutic option for individuals with severe obesity (BMI ≥ 40 kg/m^2^ or ≥35 kg/m^2^ with comorbidities) who have failed conservative management. Procedures such as Roux-en-Y gastric bypass and sleeve gastrectomy alter gastrointestinal anatomy, promoting weight loss through restriction, malabsorption, and hormonal modulation (e.g., increased GLP-1 secretion). Bariatric surgery improves obesity-related comorbidities and enhances quality of life. However, it requires lifelong medical follow-up to address potential nutritional deficiencies and ensure sustainable outcomes.

## 1. Introduction

Obesity results from a combination of behavioral, psychosocial, and environmental factors, including overnutrition, poor eating habits, physical inactivity, and genetic predispositions [1]. Additionally, a significant number of those with obesity have disordered eating behaviors linked to underlying psychological issues [1,2]. Given these factors, a thorough analysis of each patient’s individual background is essential.

As reported by the World Health Organization (WHO), in 2022, 1 in 8 people worldwide were obese, and 2.5 billion adults were overweight. These figures represent 43% of adults being overweight and 16% obese. Additionally, 37 million children under the age of 5 were categorized as overweight, while more than 390 million children and adolescents between 5 and 19 years old were reported as overweight, with 160 million of them classified as obese [3].

Current projections suggest that by 2035, over 750 million children aged 5–19 years will be overweight or obese, representing nearly two out of every five children globally. Most of these children are expected to reside in middle-income countries [4].

Obesity is a complex, chronic condition characterized by excessive fat accumulation, with excess weight being a direct consequence. This condition increases the risk of various health issues, including type 2 diabetes, cardiovascular diseases, bone and reproductive health problems, certain cancers, and impacts on quality of life, such as sleep disturbances, mobility issues, and self-image concerns.

In 2019, a BMI exceeding the obesity threshold value contributed to approximately 5 million deaths due to its association with non-communicable diseases (NCDs), including cardiovascular diseases, diabetes, cancers, neurological disorders, chronic respiratory diseases, and digestive disorders. The diagnosis of overweight and obesity is typically determined through the calculation of body mass index (BMI): weight (kg) divided by height^2^ (m^2^), supplemented by additional measurements such as waist circumference [4].

## 2. Motivation

Bariatric surgery is one of the most effective interventions for achieving significant and lasting weight loss in individuals with obesity. However, the success of the procedure depends not only on the surgery itself but also on the changes in eating behaviors that occur both before and after the operation. To improve patient outcomes and guide healthcare providers in pre- and post-surgical care, a thorough understanding of these behavioral changes is essential. Several key reasons underscore the importance of reviewing eating behaviors in this context.

While bariatric surgery can lead to substantial weight loss, maintaining this weight loss over time is closely tied to modifications in eating habits. Understanding how eating patterns evolve after surgery is crucial for preventing weight regain, a common challenge in the long term. Furthermore, eating behaviors are strongly influenced by psychological factors. Gaining insight into how patients adapt to new eating habits, manage emotional eating, and cope with physical changes provides valuable information about the psychological and physiological challenges they face post-surgery.

Pre-operative eating behaviors, such as binge eating or emotional eating, can have a significant impact on post-surgery outcomes. A comprehensive review can highlight the importance of addressing these behaviors prior to surgery to improve the chances of long-term success. Additionally, common post-surgery eating patterns, such as smaller portion sizes, food intolerances, and changes in food preferences, can help in developing personalized dietary plans and interventions for patients.

Changes in eating habits following bariatric surgery also have a considerable effect on a patient’s quality of life. A review of existing literature can explore how these adjustments impact mental health, social interactions, and overall well-being. Although considerable research has been conducted on bariatric surgery outcomes, there remain gaps in understanding the changes in eating behaviors both before and after the procedure. Reviewing existing studies can help consolidate current knowledge, identify trends, and reveal areas that require further exploration.

By examining the available evidence on eating behaviors, healthcare providers can improve pre- and post-operative care strategies, including offering enhanced psychological support, nutritional counseling, and long-term follow-up. The goal of this review is to deepen clinical understanding, improve patient care, and close the knowledge gaps regarding eating behaviors in bariatric patients. This knowledge is crucial for maximizing the benefits of surgery and reducing the risk of complications or weight regain.

## 3. Materials and Methods

### 3.1. Electronic Search Strategy

Taking these factors into account, a search was performed on PubMed using the following keywords: “obesity”, “eating behavior”, “overweight and obesity—causes and risk factors”, “eating desorders”, “bariatric surgery”, “eating behaviour and bariatric surgery”, “weight loss”, ”eating issues”, “obesity in adults/childrens”, “indication/contraindications for MBS”, “mass-media and eating behavior”, and “type/criteria for binge eating disorder”.

### 3.2. Study Selection

The search was limited to studies published in English in the last 10 years, OMS information about obesity (https://www.who.int/news-room/fact-sheets/detail/obesity-and-overweight (accessed on 15 November 2024)), *Obesity Atlas* 2024 (https://data.worldobesity.org/publications/WOF-Obesity-Atlas-v7.pdf (accessed on 15 November 2024)), and books about eating disorders.

The exclusion criteria were the patients with obesity and weight loss with diet or with medication treatment, intermittent fasting diet, and articles that did not address the topic studied in detail.

**Figure 1:**
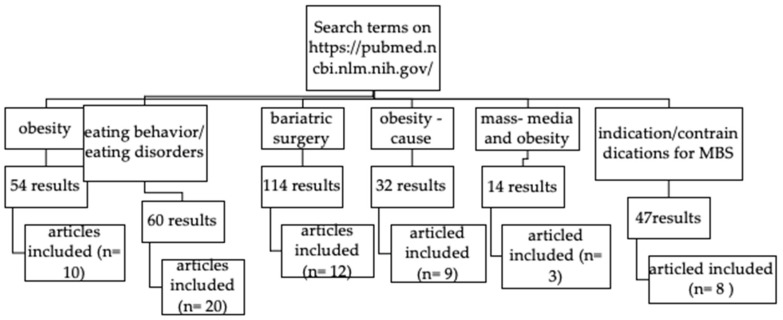
Flow diagram showing the selection process.

Figure 1 highlights the screening and selection process regarding the articles included in this review.

### 3.3. Study Appraisal

The screening process was conducted by three independent reviewers (I.M.A., B.M.C., M.M.) in duplicate. Full texts of the articles were carefully examined. Disagreements that appeared during the data abstraction phase were resolved through discussions between the reviewers. If an agreement could not be reached, a fourth reviewer (A.O.) made the final decision.

## 4. Obesity in Adults vs. Children and Adolescents

BMI categories for defining obesity differ by age and gender, particularly for infants, children, and adolescents. In adults, the WHO defines overweight as a BMI of 25 kg/m^2^ or greater and obesity as a BMI of 30 kg/m^2^ or greater. In children, however, age must be considered when determining overweight and obesity. For children over 2 years old, obesity is defined by BMI values that are higher than those of the reference population, which includes children of the same age and sex from eight different countries globally, according to the WHO.

Childhood and adolescent obesity have significant implications for health, increasing the risk of developing various non-communicable diseases (NCDs) such as type 2 diabetes and cardiovascular diseases. Obesity during these formative years also has adverse psychosocial effects, influencing school performance and quality of life and contributing to stigma, discrimination, and bullying. Children who are obese are at a higher risk of remaining obese in adulthood and are more likely to develop NCDs later in life.

Overweight and obesity result from an imbalance between energy intake (through diet) and energy expenditure (through physical activity). In most cases, obesity is a multifactorial disease, influenced by obesogenic environments, psychosocial factors, and genetic conditions. In some cases, specific etiological factors, such as medications, diseases, immobility, iatrogenic procedures, or genetic syndromes, may be identified.

The obesogenic environment that increases the likelihood of obesity includes factors such as limited access to healthy foods at affordable prices, a lack of opportunities for moderate physical activity in daily life, and the pervasive marketing of high-calorie, unhealthy foods. Additionally, the absence of effective health policies and a lack of a responsive healthcare system to identify early weight gain and fat deposition exacerbate the progression toward obesity [4].

## 5. Bariatric Surgery

Bariatric surgery is one of the most effective treatments for obesity, leading to significant weight loss (up to 60% excess weight loss post-operatively) over a period of 3–5 years, compared to only a 0–3% weight reduction with lifestyle interventions [5,6]. Although the precise mechanisms are not fully understood, post-operative neurohormonal changes are believed to play a crucial role in sustaining weight loss, particularly by promoting hunger control and increasing satiety after meals. This is especially evident following procedures like sleeve gastrectomy (SG) and Roux-en-Y gastric bypass (RYGB). When compared to pharmaceutical interventions targeting hunger and satiety, which result in a mean weight loss of just 3–8% at 12 months [7], bariatric surgery appears to have more profound effects on eating behavior. This is further supported by reports of changes in food preferences, such as a reduced craving for sweet or fatty foods and diminished food cravings after surgery [8,9].

Bariatric surgery encompasses several types of procedures, including Roux-en-Y gastric bypass (RYGB) [10], sleeve gastrectomy (SG), one-anastomosis gastric bypass (OAGB), biliopancreatic diversion with duodenal switch (DS), single-anastomosis duodeno-ileal with sleeve (SADI-S), laparoscopic gastric plication, endoscopic gastric balloon procedures, and endoscopic sleeve gastroplasty. The choice of procedure depends on various factors, such as the patient’s preferences, body mass index (BMI), medical history, and overall health. Broadly, these procedures can be categorized as restrictive (limiting the volume of the digestive tract, typically the stomach), malabsorptive (reducing the absorption of calories in the digestive tract), or a combination of both. Over the past three decades, certain procedures have become less common or been replaced by newer techniques with fewer complications [11].

There is no upper age limit for patients who may benefit from metabolic and bariatric surgery (MBS). Older individuals should be thoroughly assessed, with particular attention to co-morbidities and overall frailty [12].

### 5.1. Indications for Adolescent

- BMI ≥ 35 kg/m^2^ or ≥120% of the 95th percentile, accompanied by clinically significant co-morbidities such as obstructive sleep apnea (AHI > 5), type 2 diabetes (T2D), non-alcoholic steatohepatitis (NASH), Blount’s disease (tibia vara), slipped capital femoral epiphysis (SCFE), gastroesophageal reflux disease (GERD), or hypertension.
- BMI ≥ 40 kg/m^2^ or ≥140% of the 95th percentile, whichever is lower.

### 5.2. Multidisciplinary Assessment

Evaluation of the patient’s and family’s ability and motivation to adhere to pre-operative and post-operative treatment plans, including consistent use of micronutrient supplements.

### 5.3. Contraindications for MBS

- A medically correctable etiology of obesity.
- Active substance use disorder within the past year.
- Medical, psychiatric, psychosocial, or cognitive conditions that impair adherence to dietary and medication regimens post-surgery.
- Current pregnancy or planned pregnancy within 12 to 18 months following surgery.

### 5.4. Additional Considerations

- Unstable family dynamics, eating disorders, mental health conditions, or past trauma are not absolute contraindications to MBS but should be managed and optimized pre-operatively and peri-operatively.
- Mental health conditions, excluding active psychosis, suicidality, or substance abuse, are not contraindications for surgery. Evidence-based psychotherapeutic interventions, including cognitive–behavioral therapy, family-based therapy, and executive skill training, can aid in managing binge eating, loss of control (LOC) eating, and improving post-operative outcomes. Sleeve gastrectomy (SG) and Roux-en-Y gastric bypass (RYGB) are considered both safe and effective in treating severe obesity in adolescents.

MBS does not adversely affect pubertal development or linear growth. Therefore, neither a specific Tanner stage nor bone age should be mandated as a prerequisite for surgery [13].

### 5.5. Multidisciplinary Evaluation and Surgical Decision-Making for MBS

Candidates for MBS should undergo a comprehensive evaluation by a multidisciplinary team comprising medical, surgical, psychiatric, and nutritional specialists. This approach acknowledges the multifaceted nature of obesity and facilitates a thorough risk–benefit assessment. Additionally, such collaboration enhances the patient’s understanding of the lifelong lifestyle modifications required post-surgery, leveraging the expertise of diverse healthcare providers.

Engagement with a multidisciplinary team enables the optimization of modifiable risk factors, aiming to minimize peri-operative complications and enhance surgical outcomes. The ultimate determination of surgical readiness, however, rests with the operating surgeon.

### 5.6. Procedure Selection and Clinical Considerations

Globally, sleeve gastrectomy (SG) has emerged as the most frequently performed MBS procedure. Experts agree that SG is particularly suitable for high-risk patients, pediatric populations, and older adults (>65 years). However, SG may be less appropriate for individuals with obesity-related complications such as uncontrolled type 2 diabetes mellitus (T2DM), gastroesophageal reflux disease (GERD), or non-alcoholic steatohepatitis (NASH).

For patients with a BMI ≥ 50 kg/m^2^, SG was the preferred procedure in 66.7% of cases, although no universal consensus was reached. Procedures such as biliopancreatic diversion with duodenal switch (BPD-DS) and sleeve gastrectomy with single-anastomosis duodenoileostomy (SADI) were considered suitable for patients with a BMI > 50 kg/m^2^ and severe or uncontrolled diabetes. However, patients undergoing SADI require lifelong nutritional monitoring and supplementation to mitigate long-term risks associated with malabsorption [14].

The evaluation process for bariatric surgery includes a thorough review of the medical history, a detailed clinical interview, and psychological assessment. The psychological evaluation should be integrated into a multidisciplinary approach designed to systematically assess the risks and benefits of surgery for each patient. For patients with eating disorders, it is essential to emphasize that bariatric surgery alone will not resolve maladaptive eating behaviors. Sustainable weight loss requires ongoing efforts to modify lifestyle habits, including dietary practices and physical activity, as part of a comprehensive, long-term management plan [15].

## 6. Eating Behavior

Understanding the motivation behind eating and the factors influencing food choices is essential in addressing the growing epidemics of obesity, diabetes, and cardiovascular diseases, as diet plays a crucial role in the development and treatment of these conditions. Eating behavior is a complex interaction of physiological, psychological, social, and genetic factors that affect meal timing, portion sizes, food preferences, and food selection. Research into areas like taste genetics, food preferences, pathological eating behaviors, meal sizes, and meal choices is rapidly expanding our knowledge of how and why we eat. Recently, neural imaging techniques, particularly functional magnetic resonance imaging (FMRI), have proven effective in studying eating behavior and its genetic basis in fascinating new ways [12]. Obesity can be seen as both a consequence of eating behavior and a condition influenced by genetic, psychological, and environmental factors [13].

### 6.1. The Influence of Heredity, Family History, and Other Risk Factors

Several factors are identified as risk determinants for the onset of obesity and eating disorders (EDs). A family history of obesity is considered a major risk factor for developing obesity, attributed to significant hereditary transmission patterns [15]. Consistent with this, data from the Collaborative Psychiatric Epidemiology Surveys (CEPES) [16] have demonstrated that individuals with obesity face an elevated lifetime risk of developing EDs compared to individuals with normal weight. Additionally, a family history of binge eating disorder (BED) has been recognized as a notable risk factor for the development of obesity in adulthood [17]. Furthermore, familial obesity is linked to both BED and bulimia nervosa (BN) [18], suggesting that genetic and environmental factors within families may predispose individuals to the development of these disorders, potentially influencing their susceptibility to both disordered eating behaviors and obesity later in life [15].

Genetic factors, particularly variations in genes associated with fat mass and obesity (e.g., FTO), expressed in the arcuate nucleus of the hypothalamus—a central area in appetite regulation—have consistently been associated with obesity [19]. Other candidate genes, such as melanocortin-4 receptor (MC4R) and brain-derived neurotrophic factor (BDNF), have also been implicated in increasing susceptibility to BED in adults [20]. These findings support the hypothesis that certain genetic variations linked to excessive food intake may overlap with those predisposing individuals to overweight and obesity [21]. Recent studies also indicate genetic correlations between anorexia nervosa (AN) and metabolic traits, which have led to the reconsideration of AN as a “metabo-psychiatric disorder” [22].

Psychological traits at the individual level have also been identified as risk factors for both EDs and obesity. Heightened reward dependence, impaired cognitive control, mood dysregulation, and difficulties in emotional regulation are significant factors contributing to binge eating episodes in individuals with obesity [23]. Furthermore, personality traits such as harm avoidance (i.e., the tendency to react strongly to adverse stimuli) and low self-directedness (i.e., the ability to adapt behaviors to achieve personal goals) have been noted as specific risk factors for EDs, particularly BED and BN, as well as for obesity [18,24]. Negative self-attitudes, such as low self-esteem, negative self-evaluation, and high body dissatisfaction, are also critical risk factors for BED [25].

Environmental factors also play a crucial role in the development of obesity and EDs. Childhood adversities, including emotional or physical abuse or sexual trauma, can significantly impact an individual’s developmental trajectory, thereby increasing the risk for both obesity and EDs such as BED [24,26]. Other influential environmental factors include dietary patterns, media exposure, body image dissatisfaction, and weight-related bullying [17]. For instance, children with obesity are at an increased risk of being bullied in school, and individuals with a history of aggression have been found to have a heightened risk of developing EDs, particularly BED and BN [27].

Early identification of at-risk individuals, along with the implementation of preventive strategies, holds potential for reducing the burden of these conditions [28]. Identifying genetic markers for early detection of children predisposed to obesity could enhance strategies for managing eating behaviors in the future. Thus, current research efforts should prioritize the identification of biomarkers that indicate susceptibility to obesity and EDs, along with their shared risk factors, to facilitate early intervention and prevention [29].

Food addiction (FA) has garnered increasing attention as a factor associated with the development and maintenance of binge eating spectrum disorder (BSD) and obesity [30,31]. According to the FA model, the consumption of highly palatable foods—particularly ultra-processed foods rich in sugar, salt, and saturated fats—can lead to addictive behaviors, as these foods are particularly rewarding and can stimulate compulsive eating. The prevalence of FA has been reported to be 15.2% among adults in non-clinical populations and 15% in children and adolescents, with rates ranging from 15% to 25% in individuals with obesity [32]. Among individuals with EDs, FA is particularly prevalent, with rates of 60% in anorexia nervosa (AN), 81.5% in bulimia nervosa (BN), and 76.9% in BED [28,33]. FA is correlated with elevated ED symptomatology, general psychopathology, and poor treatment outcomes [16,34]. Additionally, FA is linked to negative urgency and low perseverance, personality traits commonly associated with emotional eating in individuals with obesity and EDs [35,36,37]. Despite the rarity of transitions from AN to BN or BED, higher FA rates in AN patients may indicate an increased risk of losing control over food intake [38]. Moreover, FA has been identified as a predictor of treatment outcomes in EDs, while it may also influence the extent of weight loss after bariatric surgery in patients with obesity [39].

The management of individuals with comorbid obesity and EDs requires a careful, nuanced approach to treatment planning [40]. While the primary goals for treating BN and BED include reducing or eliminating excessive food consumption and improving associated ED and general psychopathological symptoms, treatment objectives related to weight—particularly in BED—remain a topic of debate [41]. For obesity, the main therapeutic goals are weight loss and the maintenance of long-term weight reduction [42]. However, weight loss may often be the primary motivation for individuals seeking treatment for obesity, and some may be unaware of the presence of a comorbid ED [43]. Despite the lack of clear empirical evidence, there is concern that restrictive dietary interventions (e.g., as part of a lifestyle modification program) may exacerbate overeating behaviors or induce maladaptive eating patterns in individuals with comorbid conditions. As such, certain international treatment guidelines prioritize ED-specific treatment goals over weight management goals for patients with comorbid obesity and BED [40,44]. These guidelines recommend psychotherapy as the first-line treatment for BED, followed by interventions targeting weight loss [45]. However, there is a notable absence of cross-references between most international treatment guidelines for EDs and obesity, limiting clinical guidance for these individuals [44].

### 6.2. The Influence of Mass Media on Eating Behavior

There is substantial evidence indicating that the marketing of unhealthy foods has a direct and detrimental impact on individuals across all age groups—children, adolescents, and adults—affecting their food preferences, choices, and consumption patterns. Numerous studies have identified food marketing as a significant determinant of dietary choices. Statistically significant associations have been observed between exposure to unhealthy food and beverage marketing via social media and increased consumption of such products, as well as weight gain [45].

Consequently, this form of marketing constitutes a recognized risk factor in the development of overweight and obesity. Research by Yuanqi Gu et al. further highlights a correlation between following unhealthy food and beverage brands on social media and elevated obesity rates in the United States [46]. The sharp increase in social media food advertisements over the past decade [47], coupled with the intensifying promotion of fast-food brands and sugary beverages on these platforms, is especially alarming [48]. Evidence suggests that exposure to such advertising is strongly linked to increased consumption of fast foods and high-calorie sugary drinks among youth [49].

Despite this significant public health issue, existing studies underscore the absence of comprehensive regulatory frameworks to curb the promotion of unhealthy foods on major social media platforms [50]. Gu et al.’s findings emphasize the urgent need for robust, integrated policies aimed at restricting the digital marketing of unhealthy food products [46].

Beyond current initiatives to encourage healthier food choices, innovative approaches are essential to promote positive dietary behaviors. Leveraging marketing strategies to endorse healthy foods could play a transformative role in improving dietary habits among both children and adults, fostering a preference for nutrient-dense foods and diverse food groups [51].

## 7. Pre- and Post-Operative Eating Behavior

Establishing a robust, long-term post-operative follow-up system is critical, along with ensuring optimal nutritional and behavioral management during both pre-operative and post-operative periods. Patients with morbid obesity frequently exhibit maladaptive eating behaviors—including irregular lifestyle patterns, heightened hunger sensations, specific food preferences, and emotional eating [52,53]—that may hinder the effectiveness of dietary interventions or bariatric surgery-induced weight loss. However, the specific impact of metabolic and bariatric surgery (MBS) on these behaviors, and their relationship with post-operative weight loss outcomes, remains insufficiently understood [54].

The prevalence of MBS among patients with severe obesity has risen significantly, particularly in East Asian countries such as Japan [55,56], due to the increasing correlation between obesity and type 2 diabetes. While the benefits of MBS, including substantial weight loss and improvement in obesity-related comorbidities like diabetes, are well-documented, individual outcomes vary considerably [57,58] Suboptimal results are often associated with mental health conditions, including anxiety and depression, particularly in cases where weight loss expectations are not met [6].

Currently, bariatric surgery represents the most effective long-term intervention for morbid obesity [7,8], with patients achieving up to 60% excess weight loss post-operatively and 85% maintaining these results long-term [9]. Nevertheless, a subset of patients experiences either suboptimal weight loss [10] or significant weight regain [59]. Studies suggest that patients adhering to balanced nutrition and engaging in regular physical activity exhibit superior weight loss outcomes [9,60]. Beneficial eating behaviors include compliance with healthcare professionals [61] dietary recommendations and controlled food impulses [62]. Conversely, disordered eating patterns, such as binge eating, grazing, night eating syndrome, and emotional eating, are linked to poorer outcomes [63,64,65,66,67].

Despite the overall efficacy of bariatric surgery in fostering sustained weight loss and improved health [68,69], some individuals regain a significant proportion of the weight lost [70]. The prevalence of weight regain varies [71], with approximately 20–24% of patients regaining at least 15% of their lowest post-operative weight within five years [69,72]. The multifactorial etiology of weight regain includes disordered eating behaviors (e.g., loss of control over eating, snacking, emotional eating) [73], psychological factors (e.g., depression, anxiety), anatomical complications, sedentary lifestyles, genetic predispositions [74], and physiological changes affecting appetite regulation, such as alterations in gut hormones [75,76].

Post-surgical improvements in eating behaviors are common, with reductions in appetite and smaller portion sizes reported even a decade after surgery [77,78]. Patients tend to shift towards healthier food choices, favoring fruits and vegetables while reducing intake of sugary and fatty foods, potentially due to altered taste and olfactory perceptions [79,80,81]. Nonetheless, adopting and maintaining eating patterns conducive to sustained weight loss remains challenging [82,83]. Consumption of energy-dense foods, sugary snacks, sugar-sweetened beverages, and alcohol, as well as disordered behaviors like grazing, snacking, and stress-induced emotional eating, are strongly associated with weight regain [77,78,84].

Moreover, emerging evidence suggests that hedonic hunger and disinhibition around food contribute to poorer outcomes in individuals with suboptimal weight loss trajectories [60]. Understanding patients’ perspectives on eating patterns and behaviors during weight recovery is essential for tailoring interventions [85]. Notably, night eating behaviors and food addiction-related patterns have been associated with diminished surgical outcomes. However, existing research frequently relies on self-reported data rather than structured, empirically validated diagnostic methodologies. Furthermore, pre-operative assessments may underestimate the prevalence of disordered eating behaviors due to underreporting by patients seeking surgical approval [86,87].

Future studies should prioritize robust methodologies, including independent and structured diagnostic interviews, to accurately capture the complexities of eating behaviors and their influence on weight management following bariatric surgery [88,89,90,91].

### 7.1. Pre-Surgery Binge Eating and Binge Eating Disorder

According to the Diagnostic and Statistical Manual of Mental Disorders, Fifth Edition (DSM-5) [92], binge eating disorder (BED) is characterized by the recurrent consumption of an unusually large quantity of food within a discrete period (approximately 2 h), accompanied by a perceived loss of control (LOC) over eating. This sense of LOC refers to an inability to regulate or halt food intake during binge episodes.

BED represents the most prevalent eating disorder among candidates for bariatric surgery. The Longitudinal Assessment of Bariatric Surgery (LABS) study reported that approximately 10% of bariatric surgery candidates fulfill the full diagnostic criteria for BED, a notable contrast to the general population prevalence of 1.2% [93]. It is worth noting that these rates may underestimate true prevalence, as bariatric candidates often underreport or downplay symptoms to avoid disqualification from surgery [93]. Additionally, many individuals with BED seek treatment for obesity rather than for disordered eating behaviors or the eating disorder itself.

To meet the DSM-5 diagnostic threshold for mild BED, individuals must engage in binge eating episodes at least once per week over the preceding 3 months, with such behaviors causing clinically significant distress. Among bariatric candidates, BED severity has been positively correlated with both the degree of obesity and the presence of psychopathology [94]. Specifically, the perceived LOC during binge episodes has been associated with heightened psychological distress [95] and more severe psychopathology, including elevated rates of depressive and anxiety disorders [96]. Furthermore, individuals with BED are more likely to exhibit additional maladaptive eating behaviors [94].

### 7.2. Research Criteria for Binge Eating Disorder

A. Recurrent episodes of binge eating. A binge eating episode is characterized by the following:
  1. Eating, within a short period of time (e.g., 2 h), an amount of food that is larger than what most people would consume in a similar period under similar circumstances.
  2. A feeling of lack of control over eating during the episodes (e.g., a sense that one cannot stop eating or control what or how much they are eating).
B. Binge eating episodes are associated with three (or more) of the following:
  1. Eating much more rapidly than normal.
  2. Eating until feeling uncomfortably full.
  3. Eating large quantities of food when not physically hungry.
  4. Eating alone because of feeling embarrassed by how much one is eating.
  5. Feeling disgusted with oneself, depressed, or very guilty after overeating.
C. Marked distress related to binge eating is present.
D. Binge eating occurs, on average, at least 2 days per week for 6 months.
E. The binge eating is not associated with the regular use of inappropriate compensatory behaviors (e.g., purging, fasting, excessive exercise) and does not occur exclusively during the course of anorexia nervosa or bulimia nervosa.

Currently, there is no standardized protocol for conducting pre-surgical psychological assessments, which may result in biased estimates of binge eating disorder (BED) prevalence and associated psychopathology [97]. The use of validated eating disorder assessment tools can help address this limitation by evaluating cognitive restraint over food intake, loss of control (LOC) eating, disinhibited eating, and hunger.

A growing area of research has focused on whether pre-surgical binge eating, or BED, predicts suboptimal weight loss outcomes following bariatric surgery. While numerous studies have been conducted, most have found no significant correlation between these factors, with only a minority suggesting an association between BED and less favorable weight loss outcomes. Consequently, the presence of binge eating, or BED, should not be regarded as a contraindication for bariatric surgery [98].

Nonetheless, it is crucial to closely monitor individuals with pre-surgical binge eating or BED in the post-operative period, as evidence indicates that disordered eating behaviors may re-emerge following surgery. This underscores the importance of comprehensive post-operative care and follow-up for this patient population [99,100,101].

### 7.3. Types of BED

Picking and nibbling (P&N) is characterized by unplanned, repetitive eating episodes occurring between main meals and snacks, with an indeterminate initial quantity of food to be consumed [102]. The term is frequently used interchangeably with ‘grazing’ [92]. P&N, or grazing behaviors, are prevalent among individuals with eating disorders, such as anorexia nervosa (AN), bulimia nervosa (BN), and binge eating disorder (BED) [102]. The classification of P&N as a disordered or maladaptive eating pattern remains ambiguous, particularly in the context of bariatric surgery candidates, who may adopt this behavior as a compensatory strategy to limit food intake in efforts to lose weight [102]. Evidence suggests that pre-operative grazing behaviors are associated with an increased likelihood of persisting post-operatively, thereby adversely affecting weight loss outcomes. Consequently, these patients may benefit from targeted interventions or structured nutritional programs aimed at optimizing food intake regulation [99].

Night Eating Syndrome (NES) is defined by a normal sleep–wake cycle accompanied by recurrent episodes of nocturnal eating, characterized by awakening from sleep to consume food (nocturnal ingestion) and excessive evening food intake, exceeding 25% of total daily caloric consumption. Evening hyperphagia occurs at least twice per week [103,104] and is indicative of a delayed circadian pattern of food intake [89].

For an NES diagnosis, individuals must demonstrate awareness of their eating behaviors—distinguishing NES from parasomnias, where recollection of eating episodes is minimal or absent. Additionally, the behavior must be associated with significant psychological distress and should not be more accurately classified as another eating disorder, such as binge eating disorder (BED).

In individuals with NES, the quantity of food consumed is typically less than what is observed during a binge eating episode [105]. Notably, approximately 15–20% of individuals with obesity meet the diagnostic criteria for both NES and BED [99,105]. Studies indicate that although the prevalence of NES is rising, about 17% of bariatric surgery candidates fulfill the criteria for this condition [98,105].

### 7.4. Psychological Implications and Preparative Status 

Pre-operative eating disorders, particularly binge eating, have been linked to a range of psychopathological conditions, including elevated rates of depression [94], mood disorders [96], reduced perceived interpersonal support, increased alcohol consumption [94], and diminished health-related quality of life (HRQoL). HRQoL refers to the influence of health status on an individual’s functional capacity, encompassing both physical and mental health dimensions [106]. These domains are frequently and significantly compromised in bariatric surgery candidates [107,108], particularly among individuals with severe obesity and those with current or lifetime psychiatric disorders [109].

## 8. Feeding Issues After Bariatric Surgery

In bariatric surgery research, weight reduction has traditionally served as a primary indicator, often linked to the improvement or resolution of associated medical comorbidities, while eating behaviors have received comparatively less focus [98]. However, recent studies have highlighted that maladaptive eating patterns significantly contribute to suboptimal weight loss outcomes [110] and the occurrence of weight regain. A smaller proportion of patients may also develop post-operative eating disorders, including anorexia nervosa. Consequently, evaluating eating behaviors in the post-surgical context necessitates a comprehensive understanding of the underlying motivations that drive patients to engage in such behaviors [98].

### 8.1. Loss of Control over Eating

Although binge eating is physiologically unfeasible following bariatric surgery due to anatomical alterations, patients may continue to experience loss of control (LOC) eating and subjective episodes of overeating [111]. The highest-risk population comprises those who met the diagnostic criteria for binge eating disorder (BED) prior to surgery [100].

Conceição et al. estimated that approximately 10% of patients developed LOC episodes within two years post-surgery, with LOC reemerging as early as six months after the procedure. This behavior was associated with a range of complications [98,101], including vomiting, psychological comorbidities (e.g., depression), diminished health-related quality of life (HRQoL) [112], suboptimal short- and long-term weight loss, and increased weight regain [113,114].

As patients are unable to overeat post-operatively due to reduced gastric capacity, previous LOC eating behaviors may manifest as grazing or picking and nibbling (P&N) [110]. Consequently, inadequate weight loss and weight regain may stem not only from disinhibited eating but also from the gradual capacity to consume larger portions of food over time [115].

It is crucial to identify individuals at risk for LOC eating and monitor their eating behaviors and weight loss trajectory [116] utilizing instruments such as the Eating Disorder Examination—Bariatric Surgery Version 28, which provide more precise assessments of eating disorders following bariatric surgery.

Patients identified with LOC eating and concurrent psychopathological conditions may benefit from integrated interventions targeting both issues. However, these individuals may require additional support to maintain adherence to the post-operative diet, as evidence suggests that patients with BED or LOC eating behaviors are less likely to comply with dietary recommendations [115] and to attend follow-up appointments [117].

### 8.2. Grazing and/or P&N

Grazing and snacking (P&N)/grazing represent common problematic eating behaviors following bariatric surgery [110], affecting approximately 30% of patients in this population [109]. A key challenge in distinguishing P&N from normal eating patterns [100] is that patients are instructed to consume multiple small meals throughout the day post-operatively. P&N, however, typically occurs spontaneously, without prior planning, often leading to the intake of calorie-dense foods or beverages [97]. Grazing has been linked to post-operative weight regain [109,110], although additional research is required to further elucidate the relationship between this behavior and long-term weight outcomes.

### 8.3. Night Eating Syndrome (NES)

Currently, research on Nocturnal Eating Syndrome (NES) following bariatric surgery remains limited. However, it is generally observed to occur less frequently post-operatively [118]. A one-year follow-up study demonstrated a reduction in nocturnal eating behaviors, with prevalence decreasing from 17.1% pre-operatively to 7.8% post-operatively, though this study was confined to patients undergoing laparoscopic adjustable gastric banding (LAGB) [98]. Additionally, another study indicated an improvement in post-operative night eating symptoms in patients with pre-operative depressive mood disorders, compared to those without such mood disturbances. However, the relationship between depressive mood and NES is complex, with other factors, such as sleep disturbances, likely contributing to this predisposition [119].

It has been noted that insomnia and depressive symptoms typically improve in most patients within a short period following surgery. Currently, NES is primarily defined by the timing of eating episodes rather than their volume. Researchers hypothesize that although patients may continue engaging in nocturnal eating after surgery, the quantity consumed may be reduced [118]. Consequently, further prospective studies are needed to more comprehensively assess nocturnal eating behaviors in bariatric surgery patients [110].

### 8.4. Associations with Psychopathology and Quality of Life

Health-related quality of life (HRQoL) generally improves in the early post-operative period, encompassing both physical and mental health domains [93]. For instance, studies documenting long-term improvements in mental HRQoL have indicated that these improvements were primarily driven by weight loss but tended to diminish over time [120,121]. In patients exhibiting significant eating disorder symptoms post-surgery, binge eating disorder (BED) was associated with lower HRQoL and with frequent grazing behaviors occurring two or more times per week [110]. A similar trend was observed in another long-term follow-up study, where the presence of binge eating negatively impacted mental HRQoL nearly 14 years after surgery [122].

Thus, while initial positive changes in HRQoL and eating behaviors are commonly observed following bariatric surgery, there appears to be considerable variability in outcomes over time. In some studies, these outcomes were influenced by the type of surgery performed and were related to the extent of post-surgical weight loss [123]. This underscores the importance of long-term monitoring of eating behaviors and dietary patterns in this patient population.

## 9. Weight Loss

Recent studies suggest that disordered and problematic eating behaviors generally improve after bariatric surgery; however, the recurrence or emergence of these behaviors can hinder optimal weight loss outcomes [110]. The most consistent findings indicate that post-surgical overeating, binge eating disorder (BED), loss of control (LOC), grazing, persistent snacking (P&N), and associated psychopathological symptoms are significant negative predictors of weight loss [115]. Approximately 65% of patients who experienced weight regain post-surgery reported engaging in problematic eating behaviors [109]. Consequently, ongoing intervention and monitoring are essential throughout the post-operative period to mitigate these challenges and support sustained weight loss [115].

### Eating Disorders After Bariatric Surgery

Some studies suggest that traditional eating disorders (ED), such as anorexia nervosa (AN) and bulimia nervosa (BN), may emerge in a subset of patients following bariatric surgery [117,124].

## 10. Pre-Surgery Treatment for Eating Issues

A crucial aspect of pre-operative psychological assessment is the identification of patients with significant untreated psychopathology, including binge eating disorder (BED). In such cases, appropriate therapeutic interventions, such as psychotherapy and/or pharmacological treatment, should be considered. However, BED should not be regarded as a contraindication for bariatric surgery, as some individuals experience symptom remission following the procedure and demonstrate normal post-operative progress. Furthermore, the majority of bariatric surgery candidates exhibit considerable psychological, social, and medical challenges, making prolonged delays in surgery rarely justifiable [125].

Obese individuals with BED typically present with lower self-esteem and a higher prevalence of depressive symptoms compared to their counterparts without BED. They also exhibit a greater burden of comorbid mental health disorders, particularly mood and personality disorders [126]. Individuals with BED are more prone to report disinhibited eating behaviors and have greater difficulty in accurately interpreting hunger and satiety signals [110].

## 11. Discussion

The evaluation of pediatric patients referred for metabolic and bariatric surgery requires a thorough, multidisciplinary review of longitudinal BMI trends, comorbidities, and psychosocial and physiologic factors [127]. This includes identifying contraindications such as correctable obesity causes, substance use disorders, or pregnancy while assessing the patient’s and family’s understanding of surgical risks, benefits, and necessary lifestyle adjustments [128,129]. Referrals to experienced centers do not guarantee surgery but enable comprehensive risk–benefit discussions to guide shared decision-making. For younger children, case-by-case referral to multidisciplinary obesity centers with surgical expertise ensures tailored recommendations within a collaborative framework involving the patient, family, and healthcare providers.

Eligibility for metabolic and bariatric surgery extends beyond age, requiring consideration of the patient’s physical and psychosocial needs. A comprehensive evaluation should encompass individual and social risk factors within a holistic framework. Families must receive thorough education on the benefits and risks of surgical intervention, with their preferences prioritized. According to the American Academy of Pediatrics policy, the decision to continue care with a pediatrician or specialist should rest solely with the patient and family, as appropriate [130].

Tools for assessing BED have undergone extensive validation in adults and adolescents with overweight and obesity.

Screening questionnaires can be classified into the following two main types: pure screening tools and comprehensive assessment tools. Pure screeners, such as the Sick Control One Fat Food (SCOFF) and Binge Eating Disorder Screener (BEDS), are brief instruments designed specifically to identify individuals who may require further clinical evaluation. In contrast, longer questionnaires, such as the Eating Disorder Examination Questionnaire (EDE-Q) and Eating Disorders in Obesity (EDO) questionnaire, not only serve as screeners but also assess psychopathology and aid in differentiating specific eating disorder diagnoses. When the primary purpose is to identify patients needing clinical follow-up, the use of a pure screener may be most appropriate. A validated screening tool for identifying all eating disorders (EDs) is currently unavailable. The adolescent or youth adaptations of the EDE-Q are typically recommended for screening a range of EDs. Additionally, two tools—Adolescent Binge-Eating Disorder Questionnaire (ADO-BED) and Children’s Brief Binge-Eating Questionnaire (CBBEQ)—designed to identify binge eating disorder (BED) in youth with obesity, have demonstrated high sensitivity [131].

## 12. Conclusions

Weight loss following bariatric surgery primarily results from reduced caloric intake. Factors such as altered satiety, food reward, and dietary preferences significantly contribute to this reduction. These aspects of eating behavior do not operate independently but rather interact within the same individual, exerting cumulative or synergistic effects influenced by both conditioned and unconditioned factors. However, bariatric surgery carries inherent risks, and not all individuals with obesity are eligible for or interested in undergoing this procedure. Therefore, a deeper understanding of the mechanisms underlying the success of bariatric surgery could aid in refining other treatment options for obesity. Furthermore, some patients experience suboptimal weight loss following bariatric procedures. Investigating how these individuals differ from those who achieve greater weight loss, particularly in terms of eating behavior, may enhance the effectiveness of surgical interventions.

## Data Availability

The original contributions presented in the study are included in the article, further inquiries can be directed to the corresponding authors.
